# Can multiple sclerosis as a cognitive disorder influence patients’ dreams?

**Published:** 2013

**Authors:** Abdorreza Naser Moghadasi, Mahsa Owji

**Affiliations:** Sina Multiple Sclerosis Research Center, Sina Hospital, Tehran University of Medical Sciences, Tehran, Iran

**Keywords:** Multiple Sclerosis, Cognitive, Dreams

## Abstract

Dream should be considered as a kind of cognitive ability that is formed parallel to other cognitive capabilities like language. On the other hand, multiple sclerosis (MS) is a complex disease that can involve different aspects of our cognition. Therefore, MS may influence patients’ dreams. In fact, we do not know what the importance of dream is in MS, but further studies may introduce dream and dreaming as a sign of improvement or progression in MS disease.

Multiple sclerosis (MS) is a disease that involves different areas of the brain and is accompanied by several disorders of the central nervous system. The topology of the disease and different areas of the brain that are involved during the disease may cause various changes in dreams, because dreaming has a close relation with the human brain physiology. The destruction of this physiology by a disease like MS with the ability of involving different areas of brain, may probably lead to changes in dreaming.

This topic can also lead to a discussion from another point of view that is a different interpretation of these findings. By analyzing contents of hundreds of dreams, Domhoff concluded that the content of a dream is the continuance of our daily activities.^1^ In other words, the constitutive elements of our dreams are the ones that we think about and experience during a day. Strange and fantasy dreams constitute a very low percentage of the dreams and more than 70% of them are nothing but our daily activities in wakefulness.^2^

Domhoff represents a kind of cognitive approach in the field of dream according to this considerable fact and also the evolution of dream's content in children in accordance with their growth and changes of dream due to brain lesions. According to this approach, dream is also a kind of cognitive activity that is formed parallel to other cognitive capabilities like language. Moreover, whatever brings us various cognitive understandings such as hearing and seeing during wakefulness is effective on dream production while sleeping.^3^

We know that children's dreams, unlike the adult's, are simple and as one grows his dreams become more complex. If dreaming, as Domhoff says, is a cognitive process that is formed in a child concomitant to his/her growth; a disease that can potentially cause cognitive disorders must alter the dream, too.

Now, we know the cognitive effects of MS disease. Cognitive disorders are observed in 30-70% of the patients.^4^ Domains that are more frequently involved are sustained attention, speed processing, abstract reasoning, verbal fluency, and visuospatial perception.^5^ It seems that cognitive disorder and neurologic disorder are developing independently and are not parallel.^4^

Neuroimaging studies have demonstrated that the amount of cerebral involvement and brain atrophy have a determined relation with cognitive disorder in patients suffering from MS.^4^ Thus, MS disease with these wide cognitive presentations which are compatible with neuropsychological tests and neuroimaging studies can have many influences on different cognitive domains.

According to Domhoff's theory, dreaming is a kind of cognitive process that develops in parallel with other cognitive abilities. Therefore, MS may influence our dream. Unfortunately, neurologists do not interest in dream research. However, dream and dreaming can be considered as one of the most interesting aspects of our cognition. We do not know what the importance of dream is in MS, but further studies may introduce dream and dreaming as a sign of improvement or progression in MS disease.
